# Challenges and Opportunities for T-Cell-Mediated Strategies to Eliminate HIV Reservoirs

**DOI:** 10.3389/fimmu.2015.00506

**Published:** 2015-10-02

**Authors:** Mark A. Brockman, R. Brad Jones, Zabrina L. Brumme

**Affiliations:** ^1^Faculty of Health Sciences, Simon Fraser University, Burnaby, BC, Canada; ^2^BC Centre for Excellence in HIV/AIDS, Vancouver, BC, Canada; ^3^Department of Microbiology, Immunology and Tropical Medicine, The George Washington University, Washington, DC, USA

**Keywords:** HIV latency, T cells, CTL escape, immune evasion, immune-based therapy

## Abstract

HIV’s ability to establish latent reservoirs of reactivation-competent virus is the major barrier to cure. “Shock and kill” methods consisting of latency-reversing agents (LRAs) followed by elimination of reactivating cells through cytopathic effects are under active development. However, the clinical efficacy of LRAs remains to be established. Moreover, recent studies indicate that reservoirs may not be reduced efficiently by either viral cytopathic or CD8^+^ T-cell-mediated mechanisms. In this perspective, we highlight challenges to T-cell-mediated elimination of HIV reservoirs, including characteristics of responding T cells, aspects of the cellular reservoirs, and properties of the latent virus itself. We also discuss potential strategies to overcome these challenges by targeting the antiviral activity of T cells toward appropriate viral antigens following latency.

## Introduction

Combination antiretroviral therapy (cART) durably suppresses HIV, but the virus’ ability to persist in a quiescent state within cellular reservoirs prevents its eradication from the body. cART must therefore be maintained for life. The “Berlin patient” was cured of HIV following a stem cell transplant from a CCR5Δ32 homozygous donor that repopulated his immune system with virus-resistant cells (1), indicating that eradication is possible. But, safer and scalable strategies are clearly needed.

Latent HIV-infected cells produce very low levels of viral RNA and proteins and thus remain largely hidden from cellular immunity. Reactivation induces viral protein expression and virion production, which should make cells susceptible to viral cytopathic effects and immune targeting. However, low basal reactivation rates, maintenance of latent HIV-infected cells through homeostatic proliferation (2–6), and survival of cells following reactivation (7) ensure that reservoirs persist after many years on cART (8, 9). “Shock-and-kill” methods to reactivate latent cells (“shock”) so they can be eliminated through host or viral cytopathic effects (“kill”) (10) have been proposed to achieve clinical HIV remission (“functional cure”) or reservoir elimination (“sterilizing cure”). To avoid viral spread and establishment of new reservoirs, “shock-and-kill” is conducted in the presence of cART – but clinical successes have been limited. While identification of latency-reversing agents (LRA) supports the feasibility of this approach, elimination of reactivated cells poses a major barrier (11, 12). Here, we highlight challenges in this area, including limited clinical performance of LRAs, resistance of reservoirs to host and viral cytopathic effects, dysfunction of cytotoxic T lymphocytes (CTL), and viral immune evasion mechanisms. We also discuss strategies to enhance T-cell activity and develop T-cell-based therapeutics. For a review of antibody-mediated strategies for HIV eradication, please see Lee et al. in this series (13).

## Limitations of Latency-Reversing Agents

Latently infected cells can be induced to express HIV RNA and proteins using non-specific activating agents, such as phytohemagglutinin or anti-CD3/CD28 antibodies in the case of CD4^+^ T cells, but toxicity from cellular proliferation and inflammatory cytokines precludes their use *in vivo*. Instead, multiple classes of LRAs, including histone deacetylase inhibitors (HDACi), bromodomain inhibitors, protein kinase C agonists, cytokines such as IL-2 and IL-15, and others, have been identified that induce latent cells to produce viral RNA, proteins, and virions without causing global T-cell activation [for reviews, see Ref. (14, 15)]. However, challenges remain. Due to the rarity of latent cells *in vivo* [approximately 1 per 10^6^ resting CD4^+^ T cells (16, 17)], LRA discovery generally relies on cell lines that may not reflect reservoir complexity (18). Indeed, while the major HIV reservoir is resting CD4^+^ T cells (19), HIV persists in other cell types, tissues, and anatomical compartments (20) that remain largely untested using LRAs. Moreover, since latency is maintained via numerous mechanisms, including regulation of heterochromatic structure (21) and host factors required for gene transcription (22), LRAs with distinct mechanisms of action may need to be combined to maximize reactivation frequency or magnitude (23). Combination approaches may also benefit from reduced toxicity due to lower doses of individual agents. Toward this goal, some LRAs show synergistic ability to reactivate HIV *in vitro* (24, 25).

*In vivo* disruption of HIV latency using LRAs has been difficult to achieve. Administration of gamma-chain cytokine IL-7 generated “blips” of viremia in cART-treated individuals (26); however, this may result from productively infected cells rather than from reservoir reactivation (27, 28). More recently, three HDACi have exhibited limited ability to disrupt latency *in vivo*. Specifically, elevated levels of intracellular unspliced gag RNA – but not protein – were observed following administration of vorinostat (suberoylanilide hydroxamic acid, SAHA) (29), while panobinostat (30) and romidepsin (31) produced transient low-level increases in plasma viremia. While supporting the “shock” strategy, none of these agents appreciably reduced reservoir size. Moreover, cells became refractory to HDACi treatment following serial dosing *in vivo* (32, 33). Efforts to identify LRAs (or combinations) with greater *in vivo* potency without significant toxicity thus remain paramount.

## Challenges for T-Cell-Mediated Killing of HIV Reservoirs

Cytotoxic T lymphocytes play a crucial role in containing HIV (34–36). Determinants of CTL-mediated reservoir elimination under cART, however, may be distinct from those involved in viremia control during untreated infection. For example, whereas targeting of conserved viral epitopes – where escape is impossible or confers a substantial fitness cost (37–41) – may be desirable for natural or vaccine-induced HIV control by CTL (42, 43), this may not be critical in the context of latency reactivation since immune escape mutations will not emerge under cART. In addition, while rapid CTL-mediated killing of infected cells (i.e., before progeny is produced) might be optimal during untreated infection (44–46), prevention of viral spread by cART may allow effective targeting of viral epitopes with slower presentation kinetics. Furthermore, in untreated infection, a combination of cytolytic and non-cytolytic (e.g., interferon gamma, MIP-1, or RANTES) mechanisms (47) contain HIV, but only cytolytic activity is likely to contribute to reservoir reduction. Thus, while high-avidity CTL are beneficial for natural control of infection (48–50), they may be even more crucial to eliminate reservoirs, particularly if LRAs induce only low viral antigen levels. But some lessons from natural infection remain relevant. For example, HIV elite controllers (rare individuals who spontaneously suppress HIV plasma viremia to <50 RNA copies/mL in the absence of cART) harbor significantly lower proviral DNA levels, underscoring their potential utility to inform research toward a functional cure (51).

### Resistance of reservoirs to cytopathic effects

Consistent with their longevity *in vivo*, latently infected resting CD4^+^ T cells resist host and viral cytopathic effects following reactivation. *Ex vivo* treatment of cells with SAHA had no discernable effect on replication-competent HIV load (7), highlighting the limited ability of HDACis alone to eliminate HIV (52). Inherent features of resting CD4^+^ T cells, such as enhanced expression of survival factors or changes in metabolic state (53, 54), may also enhance their resilience. Furthermore, reactivating cells express viral proteins at low levels (55, 56), which may limit virus-induced disruption of critical host cell functions and reduce the chance of a “natural” death. This would also impair viral epitope presentation by HLA class I to CTL, impairing immune-mediated clearance. Strategies to modulate cellular metabolism (53) or apoptosis (57) may hasten cell death due to viral cytopathic effects or immune-mediated killing. Differences in antigen processing among cell types permissive to HIV (58–61) that alter the sequence, kinetics, or distribution of epitopes may also have cell-type-dependent effects on CTL recognition. Research on antigen processing and presentation in reactivating cells to identify optimal CTL epitopes should be a priority.

### Poor antiviral cytotoxic T-cell activity

Cytotoxic T lymphocytes from cART-treated individuals display limited *ex vivo* cytolytic activity against latent CD4^+^ T cells reactivated using SAHA, although killing can be enhanced by restimulating CTL with viral peptides (7). This indicates that antiviral cells are present in blood, but cannot respond effectively – perhaps due to lack of perforin or granzyme expression (62, 63). Such non-reactivity may result from prolonged absence of antigen due to cART, triggering establishment of resting central memory T cells that display lower cytolytic potential, particularly in lymphoid tissues where latent HIV is likely to reside (64). Limited T-cell trafficking and/or cytolytic function in lymph nodes may also be a concern in chronic HIV-infected individuals on cART (65, 66). Moreover, CTL exhaustion, characteristic of chronic infection and manifested by induction of “immune checkpoint inhibitors” PD-1, CTLA-4, and other inhibitory receptors (67, 68), may play a role. In any case, short-term expansion may select or amplify CTL with greater reactivity. Notably, elite controllers demonstrate better ability to eliminate latent HIV-infected cells *ex vivo* (7). This is consistent with maintenance of effector memory CTL by controllers (69) and suggests that such cells may be necessary to immediately recognize reactivating targets. Antibodies that block PD-1 (i.e., nivolumab) or CTLA-4 (i.e., ipilimumab) improve *in vivo* CTL responses against tumor-derived antigens (70), and similar approaches are being tested for HIV (71). Although studies of chronic SIV-infected rhesus macaques indicated that PD-1 blockade enhanced antiviral immunity and reduced plasma viremia (72, 73), additional human trials will be critical to evaluate this strategy (74).

Unintended negative consequences of LRAs may also hinder reservoir elimination. Immunomodulatory effects of HDACis on antigen presentation and immune cell signaling have been reported (75, 76). Moreover, treatment with HDACis (romidepsin and panobinostat) at clinically relevant doses impaired CTL cytokine production and cytolytic responses toward HIV target cells (77). The effects of other LRAs on immune function have not been reported and should be assessed during pre-clinical testing.

### Viral evasion from T-cell immunity

Cytotoxic T lymphocyte killing requires recognition of peptides presented in complex with HLA class I on the infected cell surface. As such, the ability of HIV to evade CTL through mutational escape (78) and Nef-mediated downregulation of HLA class I (79) are highly relevant to reservoir elimination efforts.

HIV eludes CTL by altering the sequence of viral epitopes in a manner that is predictable based on the HLA class I alleles expressed by the host (80, 81). As immune escape (82–86) and seeding of the reservoir (87) begin in early infection, the presence of escaped epitopes in latently infected cells is a major barrier. Indeed, mutations in proviral sequences from cART-treated individuals reduced CTL recognition of these cells following reactivation (88, 89). Ongoing reservoir seeding poses additional challenges. While plasma HIV RNA sequences reflect contemporary viral forms that have survived multiple within-host immune bottlenecks, the reservoir is likely to comprise a genetically heterogeneous population reflecting multiple descendant lineages from the transmitted/founder viral strain, including extinct ones. Thus, the latent pool is likely to include escaped and non-escaped (archival) forms of the same epitope [though it has been noted that the majority of reservoirs carry some escape mutations (88)]. Eliminating such a heterogeneous target may require revitalization of CTL against non-escaped epitopes as well as elicitation or expansion of CTL capable of responding to more diverse sequences including escape mutations (11) and subdominant epitopes (90). These considerations are somewhat distinct from vaccine strategies that traditionally focus on conserved viral elements (42, 43). Importantly, in addition to improving clinical outcomes (91–93) and limiting transmission (94, 95), early cART reduces reservoir size and diversity (96–99), underscoring “seek, test, and treat” approaches to improve the odds of cure. Addressing reservoir diversity will nevertheless be important, as most individuals initiate cART after reservoirs encoding escape mutations are established.

Downregulation of HLA-A and HLA-B molecules by HIV-1 Nef represents another key CTL evasion strategy (100, 101). While no studies have explicitly examined the impact of Nef-mediated HLA-downregulation in the context of latency reversal, early expression of Nef (before Gag, Pol, and Env) (79) will presumably allow it to function similarly in reactivating cells. As such, identifying early viral epitopes presented before Nef acts (102, 103) may be useful for eradication. In contrast to untreated infection, where Gag epitopes from incoming virions can be presented to CTL prior to Nef-mediated HLA downregulation (104), the earliest viral peptides presented following reactivation will be derived from accessory/regulatory proteins (Tat, Rev, Nef) expressed by the integrated provirus. CTL targeting these proteins are not generally associated with control in untreated HIV infection (105), but may nevertheless be beneficial (106), particularly for Tat (107). Other Nef features may also be relevant. As HLA-B alleles display some resistance to Nef-mediated downregulation compared to A alleles (108), HLA-B-restricted CTL may be better able to recognize reactivating cells [though one study reported no difference when cells were restimulated *ex vivo* with a small number of A- versus B-restricted peptides (88)]. HLA-C is not downregulated by Nef (109, 110) and HLA-C expression correlates with HIV control (111); thus C-restricted epitopes may be attractive targets. In addition, Nef’s ability to downregulate CD4 may contribute to reservoir evasion from antibody-dependent cellular cytotoxicity (13, 112–114). Notably, patient-derived Nef sequences differ in their ability to downregulate HLA class I and CD4 (115, 116), and these Nef functions can be attenuated through within-host viral adaptation to CTL (117), indicating that Nef’s ability to modulate HIV latency may differ based on viral and immunogenetic factors unique to each host. Small molecule inhibitors of Nef (118) might enhance the visibility of cells following reactivation.

## Targeting Reservoir Diversity

Substantial interindividual heterogeneity in reservoir size and sequence (i.e., early versus late cART, prevalence of escape mutations) and host CTL responses (i.e., HLA type, dominant epitopes targeted, exhaustion) highlight the complexity of HIV elimination and imply that a “one-size-fits-all” approach may not be fully successful. T-cell-based therapies tailored, in part, to features of individual patients may help to move us toward approaches for HIV cure.

### Genetic characterization of the reservoir

Evaluation of reservoirs focuses mainly on quantifying proviral DNA, RNA transcripts, and viral outgrowth (119). Replicative competence is also important, although the high levels of gene-deleted or hypermutated sequences seen in latent reservoirs may contribute to inflammation (52). As such, genetic analyses of latent HIV sequences as well as host factors (i.e., HLA) (88) may pave the way for more personalized immunotherapeutic strategies: next-generation sequencing technologies will be particularly useful in this regard. At the most basic level, such approaches may identify non-mutated CTL epitopes that can serve as immune targets, analogous to use of HIV drug resistance genotyping to guide cART (120, 121). Characterization of latent HIV diversity may shed light on another key question – that of elucidating the chronology of reservoir establishment in different cell types and tissues. As CTL escape mutations are highly reproducible in terms of HIV genomic locations (41, 80, 81) and selection kinetics (84–86, 122, 123), they can provide a crude estimate of the relative age of reservoirs. While this has been examined in the context of SIV where founder viral sequence and inoculation date are known (124, 125), refined estimates of HIV reservoir age in humans may require more advanced phylogenetic approaches. The problem of dating reservoir sequences within an individual’s infection history is similar to that of dating organismal sequences of unknown age in the context of macroevolution [i.e., ancient DNA (126)] or in the case of HIV, specimens archived from historic eras (127, 128). In the latter case, heterochronous HIV sequences (i.e., those sampled from different individuals over the epidemic’s course) are used to calibrate viral evolutionary rates to calendar time using Bayesian (126) or root-to-tip regression (127) approaches, allowing the estimation of sampling times (tip-ages) for sequences of unknown age. Similarly, within-host plasma HIV RNA sequences sampled longitudinally from a given individual could be used to calibrate a host-specific HIV evolutionary rate that could be used to infer the date of establishment of individual reservoir sequences (129). Such analyses may be beneficial to retroactively investigate “partial” successes using shock-and-kill (e.g., to investigate whether the age of a reservoir predicts its potential for reactivation or CTL elimination).

### Therapeutic vaccines

Knowing that an epitope is present in the reservoir is only the first step. Although no therapeutic vaccine has succeeded in suppressing HIV viremia long-term, there is renewed interest to couple vaccines with LRAs and cART for additive effects (130). HIV persistence in tissues beyond the mucosa and lymphatic system, and strategies to enhance CTL patrol of these areas, represent major challenges. For example, T follicular helper cells located in B-cell follicles of lymph nodes may be a major reservoir (131–133); whole-body imaging of SIV-infected rhesus macaques revealed ongoing replication in the respiratory tract and lung tissue during cART (134), identifying these as potential sanctuary sites. Of note, a Phase II trial examining Tat as a therapeutic vaccine target demonstrated restoration of immune cells (including effector memory CD8^+^ T cells) and reduction of proviral DNA in blood (107); similar studies to assess other early viral protein targets (i.e., Rev and Nef) are also warranted.

Vaccine delivery methods are another consideration. Replication-competent and replication-defective viral vectors, nucleic acids, proteins, and various adjuvants have been tested in the context of HIV vaccines (135). Recently, a replication-competent simian cytomegalovirus vector expressing T-cell antigens has shown promise in a rhesus macaque model (136). While this vaccine did not generally prevent SIV infection, animals cleared viral RNA and DNA from plasma and tissues over the course of 1–2 years without cART (137). Although the mechanism of clearance is unknown, the vector’s ability to maintain effector memory CTL, including those targeting non-canonical HLA class II and HLA-E-restricted epitopes, is likely to play a role (136, 138). Regardless of the vector used, CTL elicited by a vaccine must be cytolytic and capable of trafficking to sites where HIV resides.

### T-cell-based therapies

Advances in cancer treatment, including adoptive transfer of tumor infiltrating lymphocytes and antigen-specific T-cell receptor (TCR) gene therapy (139), have reinvigorated the field of T-cell-based therapies. Cancer and HIV treatment, however, differ in key respects, risk/benefit considerations being one. Support for immunotherapeutic approaches remains strong in the context of limited life expectancies and lack of alternative therapies for certain cancers, but new HIV treatments must meet a high barrier for implementation due to the potency and safety of cART. Nevertheless, the lack of serious adverse events in recent cancer immunotherapy trials bodes well for testing such approaches for HIV cure (140–142).

Adoptive therapy using autologous virus-specific T cells is not new to HIV (143), but studies have thus far been unsuccessful. Over the past 20 years, several groups have attempted *ex vivo* expansion and reinfusion of patients’ own CTL (144–148). Limitations of these trials, including inefficient engraftment or survival of cells and lack of cART, are now addressable using optimized methods and improved treatment options. Transfer of autologous tumor infiltrating T cells is used routinely for some cancers (149, 150). Moreover, successful expansion of HIV-specific CTL from cART-treated individuals that display *ex vivo* cytolytic activity against autologous reservoir cells (89, 151) indicates that newer adoptive T-cell strategies may demonstrate improved HIV efficacy, particularly if they can be coupled with LRAs.

Gene therapy approaches represent another possible avenue. Modification of CTL to express a heterologous TCR can redirect cells toward a specific antigen. Several strategies have been employed (140, 149), including native (unmodified) TCRs, affinity-enhanced TCRs, and “chimeric” antigen receptors (CAR) that typically encode the antigen-binding domain of immunoglobulin linked to an intracellular signaling domain such as CD3zeta. These methods are being assessed for various cancers and they are in earlier stage development for HIV – with several reports demonstrating antiviral activity *in vitro* or in small animal models. By reprogramming hematopoietic stem cells to express a TCR against the HLA-A*02-restricted Gag SL9 epitope (SLYNTVATL), Kitchen et al. (152) suppressed HIV viremia and reduced proviral DNA loads in a humanized mouse model. CTL have also been engineered to express HIV-specific CARs, including those targeting HIV gp120-expressing cells (153, 154). In addition, gene therapy may allow reprogramming of other critical CTL functions, including cytotoxicity or lymphoid trafficking potential. Similarly, combining gene therapy approaches with other immune modulators (such as blockade of checkpoint inhibitors) could provide added benefits. As a note of caution, clinical trials using an affinity-enhanced TCR specific for Gag SL9 (155) were canceled when severe toxicity was observed for a similar product against melanoma tumor antigen (156). *In vivo* safety thus remains a concern, but these methods offer highly flexible strategies to target HIV reservoirs.

## Conclusion

Extraordinary progress is being made to understand the molecular mechanisms of HIV latency and to discover viral reactivation strategies. Overcoming barriers to eliminate latent cells will be critical for shock and kill strategies to succeed, and many important issues remain. What viral epitopes are presented efficiently by reactivating cells? Is antigen presentation affected by LRAs? Does Nef modulate the sensitivity of reactivating cells to CTL killing? Can therapeutic vaccines enhance reservoir targeting? Will T-cell-based therapeutics be safe and effective? Answers to these and other questions will guide future directions in this field and may ultimately determine whether we prevail in the quest to cure HIV.

## Conflict of Interest Statement

The authors declare that the research was conducted in the absence of any commercial or financial relationships that could be construed as a potential conflict of interest.

## Abbreviations

CAR, chimeric antigen receptor; cART, combination antiretroviral therapy; CTL, cytotoxic T lymphocyte; HDACi, histone deacetylase inhibitor; LRA, latency-reversing agent; SAHA, suberoylanilide hydroxamic acid; TCR, T-cell receptor.
